# Chondroitin Sulfate Proteoglycan 4 (CSPG4) as an Emerging Target for Immunotherapy to Treat Melanoma

**DOI:** 10.3390/cancers16193260

**Published:** 2024-09-25

**Authors:** Xinyi Chen, Shabana Habib, Madalina Alexandru, Jitesh Chauhan, Theodore Evan, Joanna M. Troka, Avigail Rahimi, Benjamina Esapa, Thomas J. Tull, Wen Zhe Ng, Amanda Fitzpatrick, Yin Wu, Jenny L. C. Geh, Hawys Lloyd-Hughes, Lais C. G. F. Palhares, Rebecca Adams, Heather J. Bax, Sean Whittaker, Joanna Jacków-Malinowska, Sophia N. Karagiannis

**Affiliations:** 1 St. John’s Institute of Dermatology, School of Basic & Medical Biosciences & KHP Centre for Translational Medicine, King’s College London, London SE1 9RT, UKjoanna.m.troka@kcl.ac.uk (J.M.T.); avigail.rahimi@kcl.ac.uk (A.R.); heather.bax@kcl.ac.uk (H.J.B.);; 2 Oncology Department, Guy’s and St Thomas’ Hospitals, London SE1 9RT, UK; 3 Breast Cancer Now Research Unit, School of Cancer & Pharmaceutical Sciences, King’s College London, Innovation Hub, Guy’s Hospital, London SE1 9RT, UK; 4 Peter Gorer Department of Immunobiology, Centre for Inflammation Biology and Cancer Immunology, School of Immunology and Microbial Sciences, King’s College London, London SE1 9RT, UK; 5 St John’s Institute of Dermatology, Guy’s, King’s and St. Thomas’ Hospitals NHS Foundation Trust, London SE1 9RT, UK; 6 Department of Plastic Surgery, Guy’s, King’s and St. Thomas’ Hospitals, London SE1 9RT, UK

**Keywords:** chondroitin sulfate proteoglycan 4 (CSPG4), melanoma, antibodies, antibody–drug conjugates (ADCs), immunotherapy, chimeric-antigen receptors (CAR) T cells, vaccines

## Abstract

**Simple Summary:**

Despite the emergence of several approved targeted and immune therapies for melanoma, clinical management continues to present challenges. These include resistance to clinically available treatments and lower response rates for late-stage and metastatic disease, translating to less favourable survival. Additionally, approved immunotherapies are not directed specifically against cancer cells. New treatments are therefore required to improve outcomes. One such approach entails therapeutics targeting molecules on the cancer cell surface but less frequently or lowly expressed in normal tissues. Chondroitin sulfate proteoglycan 4 (CSPG4) is a transmembrane protein overexpressed in solid tumours, including melanoma, with restricted expression in normal tissues, potentially fulfilling key criteria as a promising therapeutic target. Here, we describe the known functions of CSPG4 in healthy states and in melanoma. We review different approaches designed to activate the patient’s immune system to target CSPG4-expressing melanomas, and we discuss how such interventions could be translated into clinical practice.

**Abstract:**

Immunotherapies, including checkpoint inhibitor antibodies, have precipitated significant improvements in clinical outcomes for melanoma. However, approximately half of patients do not benefit from approved treatments. Additionally, apart from Tebentafusp, which is approved for the treatment of uveal melanoma, there is a lack of immunotherapies directly focused on melanoma cells. This is partly due to few available targets, especially those expressed on the cancer cell surface. Chondroitin sulfate proteoglycan 4 (CSPG4) is a cell surface molecule overexpressed in human melanoma, with restricted distribution and low expression in non-malignant tissues and involved in several cancer-promoting and dissemination pathways. Here, we summarize the current understanding of the expression and functional significance of CSPG4 in health and melanoma, and we outline immunotherapeutic strategies. These include monoclonal antibodies, antibody–drug conjugates (ADCs), chimeric-antigen receptor (CAR) T cells, and other strategies such as anti-idiotypic and mimotope vaccines to raise immune responses against CSPG4-expressing melanomas. Several showed promising functions in preclinical models of melanoma, yet few have reached clinical testing, and none are approved for therapeutic use. Obstacles preventing that progress include limited knowledge of CSPG4 function in human cancer and a lack of in vivo models that adequately represent patient immune responses and human melanoma biology. Despite several challenges, immunotherapy directed to CSPG4-expressing melanoma harbors significant potential to transform the treatment landscape.

## 1. Introduction

Melanoma is the most aggressive form of skin cancer, accounting for 1 in 5 skin cancer cases, and with increasing incidence rates in the last 50 years, especially in European ancestry populations [1,2]. In the UK, melanoma contributes to over 70% of skin cancer-related deaths [1,2]. Patient outcomes vary, with a >95% 5-year survival rate for early (stage 1) localised disease and 50–60% 5-year survival rates for late-stage metastatic melanoma (stage 4), despite several approved targeted pathway and checkpoint inhibitor treatment options [3] in the last 13 years. 

Two hallmarks of melanoma, which have been actively targeted with therapies, have positively influenced outcomes for patients in the last decade or so: (a) the high rate of mutations in the MAPK pathway and (b) the fact that melanoma is a highly immunogenic tumour. 

Approximately 50–60% of melanomas bearing mutations in the protein kinase BRAF are eligible for treatment with BRAF and MEK inhibitors, targeting mutant forms of BRAF (BRAFi) and MEK (MEKi) in the mitogen-activated protein kinase (MAPK) pathway to block cancer cell survival signals [4]. These drugs are now widely implemented as first-line therapies [5,6]. BRAF inhibitors can induce tumour regression in ~50% of patients with an activating mutation of the BRAF kinase [7]. Despite initial responses, intrinsic or acquired resistance to treatment often occurs within several months.

As a cancer with a significant mutational burden, melanoma is a highly immunogenic tumour that paradoxically escapes immune clearance [8,9]. Escape mechanisms include immunosuppression of the tumour microenvironment (TME) through secretion of cytokines, downregulation of tumour-associated antigens (TAAs) and tumour surface antigens (TSAs) to inhibit clearance by cytotoxic T lymphocytes (CTLs), and manipulation of immune checkpoints [8,10,11]. Checkpoint inhibitors block the regulatory functions of cell surface checkpoint molecules, such as the programmed death-1, PD-1, and its ligand PD-L1, cytotoxic T-lymphocyte-associated protein 4 (CTLA-4), and lymphocyte activation gene 3 (LAG-3). In addition to the first two immune checkpoint inhibitors (ICIs), the latter was recently approved by the U.S. Food and Drug Administration (FDA) in 2022 for the treatment of metastatic or unresectable melanoma in combination with nivolumab [12]. The success of checkpoint inhibitors demonstrates that manipulation of the immune environment can be achieved to improve clinical outcomes. Yet, there are several challenges associated with treatment. These include high toxicities, non-responsiveness in a significant proportion of patients, and the development of resistance in patients who initially respond [13,14,15]. As of yet, no reliable biomarkers exist to identify patients a priori who will not respond or develop toxicities. Fundamentally, new treatment options are required to improve outcomes and reduce the risk of treatment pauses. One such approach entails the development of therapeutics specifically targeting melanoma cells. However, there is a lack of validated targets, especially cell surface antigens that are highly expressed in cancer and less frequently or lowly expressed in normal tissues, to provide a therapeutic window for targeted treatments. 

Chondroitin sulfate proteoglycan 4 (CSPG4), also known as melanoma-associated chondroitin sulfate proteoglycan (MCSP) or neuron-glia antigen-2 (NG2) in mice and rats, is a transmembrane protein, first described over 40 years ago, which is highly expressed on human melanoma cells [16]. Based on overexpression in solid tumours such as melanoma or triple-negative breast cancer (TNBC) but restricted expression in normal tissues, CSPG4 has been proposed as a promising therapeutic target. Here, we describe what is currently known with regard to the functions of CSPG4 in health and melanoma. We review different antibody-based and immunotherapeutic approaches targeting CSPG4 in melanoma and discuss how such immune cell-targeting interventions could be refined to attain, enhance, prolong, or maintain clinical efficacy.

## 2. CSPG4 Structure, Expression and Functions in Health

CSPG4 is a single-pass type 1 transmembrane protein expressed in different tissues throughout the body, including the gastrointestinal tract, brain, and endocrine organs such as the pancreas [17]. High CSPG4 expression in healthy tissue is largely restricted to precursor or progenitor cells, whilst in terminally differentiated cells, CSPG4 expression often ceases. For instance, CSPG4 is expressed on epithelial stem cell compartments, including hair follicles and basal keratinocytes, indicating a role in skin and hair follicle development [18]. CSPG4 also presents on endothelial cells and activated pericytes but not on mature vasculature, highlighting its supporting role in early angiogenesis and vascularization [19,20]. Additionally, CSPG4 is found on chondrocytes of the articular cartilage, smooth muscle cells, brain pericytes, and neuromuscular synapse cells of human postnatal skeletal muscles. The tissue-specific expression of CSPG4, particularly in areas undergoing development, repair, or remodeling, makes it a valuable target for therapeutic intervention, especially in pathological conditions such as cancers, where increased cell proliferation and aberrant tissue growth or remodeling are reactivated [21,22].

**Figure 1:**
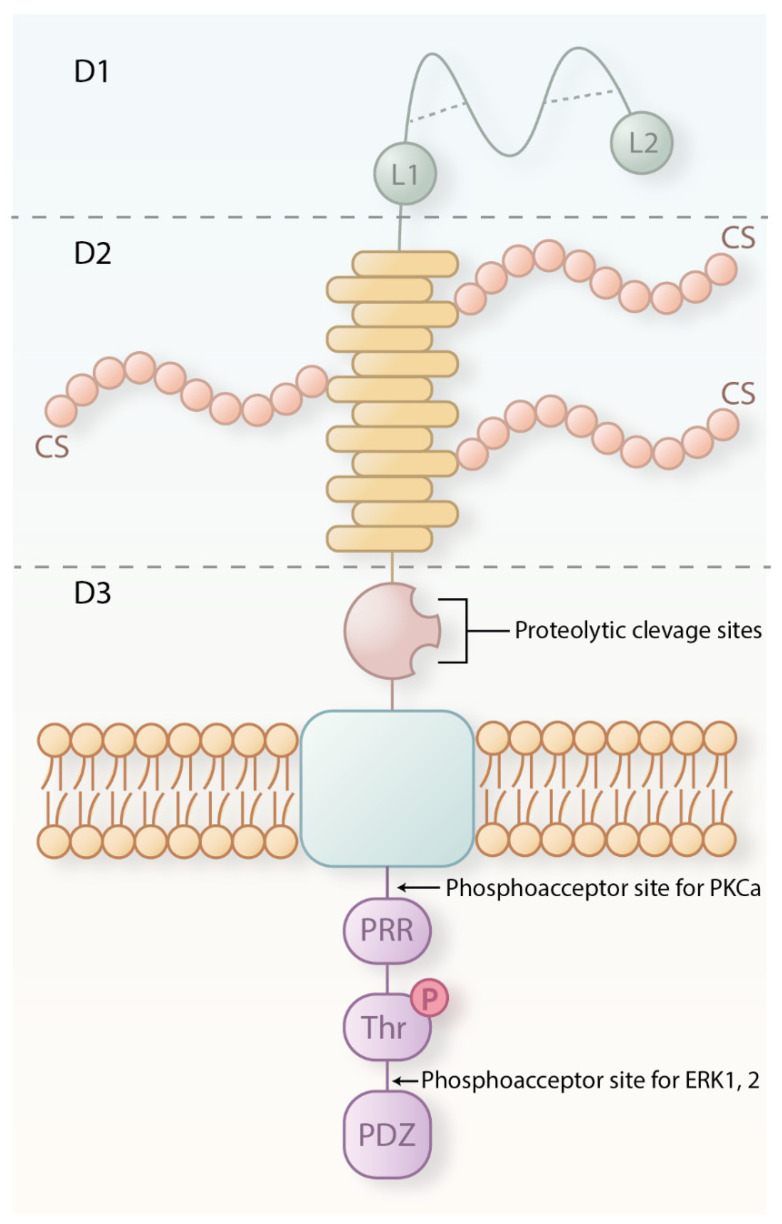
Schematic representation of CSPG4 structure and the proposed structural and functional significance of its extracellular and intracellular regions and subdomains. The extracellular region consists of three subdomains: D1, D2, and D3. D1, stabilized by disulfide bonds (dash lines), is proposed to bind laminins via an LG region (L1 and L2). D2 has 15 CSPG repeat sequences and multiple CS binding sites. D3 contains potential matrix metalloproteinase (MMP) cleavage sites. Intracellularly, key phosphorylation sites include Thr2252 (PKC-α) and Thr2310 (ERK1/2). The proline-rich region (PRR) and PDZ binding motif facilitate interactions with proteins like synthenin, MUPP1, and GRIP1.

CSPG4 in normal tissues exists as either a 250 kDa core glycoprotein or a 450 kDa chondroitin-sulphate (CS) decorated proteoglycan (Figure 1) [23]. Studies on the NG2 rat homologue have shown that CSPG4 is composed of a large extracellular domain and shorter transmembrane and cytoplasmic domains [24]. The extracellular portion of CSPG4 contains three subdomains: D1, D2, and D3. The D1 domain is the furthest from the membrane and is abundant in cysteine residues that form disulphide bonds thought to contribute to structure stability [24,25]. This region also contains two laminin-G-type domains, which are thought to be involved in mediating interactions with the extracellular matrix [23]. The middle D2 domain harbours fifteen CSPG4-specific repeats that are reported to contain potential glycosylation, chondroitin sulfate glycosaminoglycan (CS), an attachment that may facilitate interaction with α4β1 integrin, fibronectin, and matrix metalloproteinases (MMPs) integrin [26]. D3, the domain that is most proximal to the membrane, contains modified carbohydrates capable of binding lectins such as galectin-3 and integrins [27]. The cytoplasmic domain contains two threonine (Thr) phosphorylation sites. The phosphorylation of Thr2256 by protein kinase Cα (PKCα) and of Thr2314 by extracellular signal-regulated kinase (ERK) is involved in cell proliferation and motility [28]. Additionally, within the cytoplasmic domain, a proline-rich region is considered responsible for interactions with intracellular proteins [23,29], and a four-residue PDZ domain binding motif at the C-terminus can bind scaffold/adaptor proteins that contain the PDZ domain such as multi-PDZ domain protein 1 (MUPP1) [30]. Both features might have a role in connecting CSPG4 to key signalling molecules within the cytoplasm. 

**Figure 2:**
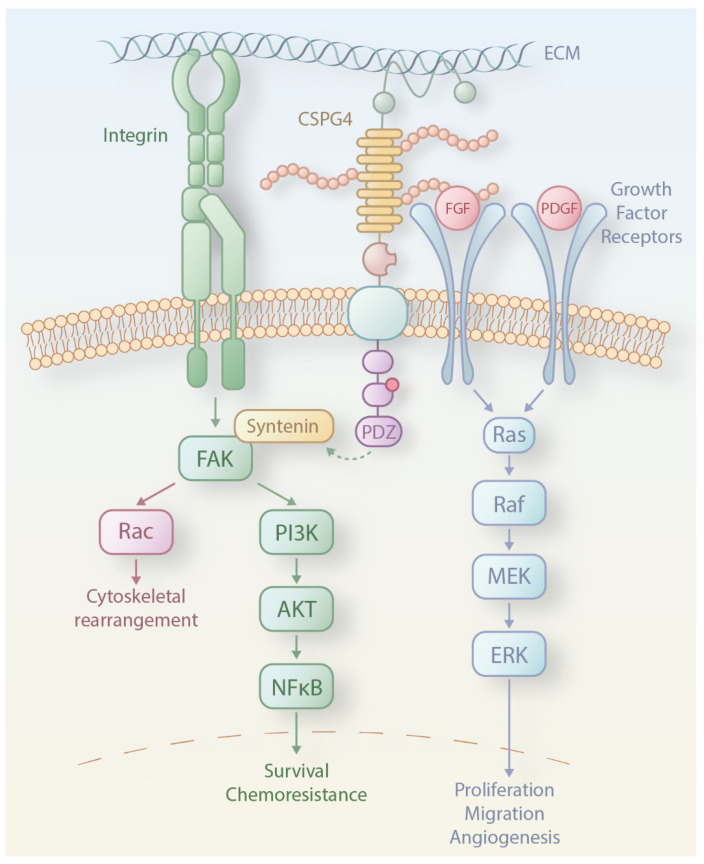
Interactions of CSPG4 with intracellular signaling pathways. CSPG4 is reported to activate two primary signaling cascades. These interactions are thought to occur through its cytoplasmic domain and two distinct pathways: the focal adhesion kinase (FAK) integrin signaling pathway and the mitogen-activated protein kinase (MAPK)/extracellular signal-regulated protein kinase (ERK) pathway. In the FAK pathway, the scaffold protein syntenin facilitates the formation of the FAK/Src complex, leading to the activation of Rac and inducing cytoskeletal rearrangement. The FAK complex also triggers PI3K phosphorylation and subsequent AKT activation, regulating NF-κB transcriptional activity and promoting survival and chemoresistance. In the MAPK/ERK pathway, together with growth factor receptors, CSPG4 activates the small GTPase RAS, which phosphorylates MEK and subsequently ERK, supporting migration, proliferation, and angiogenesis.

The structural features of CSPG4 imply a role as a mediator to potentiate the communication between the extracellular matrix and intracellular signalling pathways (Figure 2). The two pathways activated by CSPG4 are the focal adhesion kinase (FAK) pathway and, particularly pertinent in melanoma, the mitogen-activated protein kinase (MAPK) pathway. CSPG4 links to the extracellular-matrix-sensing FAK pathway through its interactions with integrins [31]. The MAPK pathway, however, can be activated in two ways: by CSPG4 binding and presentation of growth factors to adjacent cognate receptor tyrosine kinases (RTKs) or by direct activation through ERK1/2 [28,32].

Studies on knockout mice show that deletion of the mouse CSPG4 homologue NG2 is not lethal, suggesting that expression is not essential for survival [33]. One of the established physiological functions of NG2 is its involvement in angiogenesis, as it was shown to induce in vivo vascularization in an avascular corneal tissue of mice [27]. Other studies suggest involvement [34] in the formation of the human placenta [35] and in neural network regulation in mice [36]. Expression on extravillous trophoblasts suggests roles in cell differentiation and migration, while studies implicate CSPG4 in the formation and regulation of glial cells such as oligodendrocytes, epithelial keratinocyte replenishment and epidermal stem cell homeostasis [36,37]. Therefore, the evidence so far points to a likely involvement of CSPG4 in tissue development and homeostatic processes such as cell proliferation, survival, invasion, and migration. 

The mechanisms regulating the expression of CSPG4 remain insufficiently understood. There is evidence that the microenvironmental conditions such as the presence of hypoxia-inducible factors (HIFs) and inflammatory cytokines IL-1α, IFN-γ, TNF-α, and TGF-β as well as epigenetic mechanisms, certain transcription factors and microRNAs are involved in CSPG4 regulation [38].

## 3. CSPG4 Expression and Functions in Melanoma

CSPG4 expression has been identified in several malignancies, including melanoma, glioma, triple-negative breast cancer and pancreatic cancer, as well as in cancer-initiating cells (CICs) [39]. CSPG4 is reported to be expressed on the surface of 65% of primary and metastatic melanoma samples in a patient cohort from Austria. The primary melanoma subtypes with the highest expression frequency (75%) were nodular and superficial spreading melanoma, whilst the lowest expression frequency was found in lentigo maligna (12.5% of samples) [40]. Another study demonstrated overexpression of CSPG4 in several melanoma cell lines at the protein and mRNA levels and expression in human melanomas but less prominently in normal human tissues (n = 30) by immunohistochemistry and reverse-phase protein arrays [41]. Another recent study analysed CSPG4 protein expression by immunohistochemistry in melanoma samples (n = 428) and normal tissues (n = 558) and found expression in a similar proportion of melanomas (63%), a finding recapitulated by transcriptomic analyses [42]. In addition, soluble CSPG4 has been detected in the serum of both healthy individuals and melanoma patients [43]. Since 29% of patients show elevated circulating CSPG4 levels, it has been suggested that serum CSPG4 could serve as a diagnostic biomarker in melanoma [43]. Potentially, soluble CSPG4 in patient circulation may sequester anti-CSPG4 therapeutic agents, thus restricting their ability to reach and target CSPG4-expressing cancer cells in tissues.

Knock-down or blockade of CSPG4 in established tumours results in significant tumour growth inhibition in glioblastoma, breast cancer, sarcoma, and melanoma models [44,45,46], suggesting tumour-promoting roles. However, CSPG4 is not reported to have any intrinsic catalytic functions but rather serves as a scaffold protein [23], promoting cancer growth and progression through involvement in pathways regulating epithelial-to-mesenchymal transition (EMT), cell motility, and proliferation. The expression of full-length CSPG4 in human melanoma cells was shown to cause sustained activation of ERK1,2 signalling and promote cell growth and EMT. Activation of ERK1 and 2 subsequently regulates microphthalmia-associated transcription factor to enhance fibronectin expression, suppress E-cadherin expression and disruption of normal cellular adhesion [23,29,47,48]. CSPG4 inhibition by small interfering RNAs (siRNAs) in cancer cell lines was shown to lead to a reduction of ERK1,2 signalling [49]. Activation of ERK1,2 downstream of CSPG4 may promote cancer cell proliferation, angiogenesis, and migration, hence supporting tumour growth and metastasis (Figure 2). The CS groups can also interact with P-selectin on endothelial cells, thereby promoting endothelial cell activation and vascular development [50]. On the other hand, CSPG4 enhances the activation of the integrin-FAK pathway by interacting with the extracellular matrix elements and promoting integrin clustering. This may lead to cytoskeletal rearrangement, angiogenesis, and invasiveness of cancer cells [31,51]. Induction of CSPG4 expression in a CSPG4-negative human melanoma cell line enhanced integrin-mediated cell spreading as well as activation of FAK and MAPK/ERK pathways, both associated with malignant progression in melanoma [31]. One study details a decline of T-helper cell reactivity against CSPG4 in melanoma patients compared with healthy subjects, suggesting that there may be potential CSPG4-specific protection against melanoma cells in non-malignant states [52]. Taken together, those studies suggest that CSPG4 functions amplify intracellular signalling pathways that provide a growth advantage for melanoma cells. Nevertheless, all these studies have been conducted in vitro on melanoma cell lines. Further research on CSPG4-mediated tumour formation in vivo and ex vivo in human tumour explants or 3D models, such as organoids, is still necessary.

Interestingly, there is evidence that CSPG4 may contribute to treatment resistance. Yu and colleagues showed that a monoclonal antibody inhibiting CSPG4 either prevented or delayed the development of melanoma cell resistance to the BRAF inhibitor PLX4032 BRAF in vitro. The likely mechanism by which CSPG4 enhances the resistance is through activation of kinases such as FAK or ERK1/2, which were downregulated upon administration of the CSPG4 antibody [53]. Similarly, CSPG4 expression was also shown to confer resistance of melanoma cells to TNFα, doxorubicin and cisplatin treatment, most likely through activation of α3β1 integrin/PI3K signalling, which promotes cell survival [54]. A CSPG4 polymorphism was reported to predict shorter progression-free survival in patients treated with the vascular endothelial growth factor (VEGF)-targeting monoclonal antibody Bevacizumab in combination with chemotherapy [55].

The expression pattern of CSPG4 combined with putative roles in multiple oncogenic and treatment resistance-promoting pathways render this a promising target for cancer therapies that are designed to inhibit its pro-tumour functions or focus immune responses against CSPG4-expressing melanomas. 

## 4. CSPG4 and the Immune Response

Few studies have looked at the immunogenicity of CSPG4. Circulating CSPG4-reactive CD4+ T cells have been detected in healthy individuals and patients with melanoma. However, no significant correlations between T cell responses against an HLA-DR-presented CSPG4 peptide through IFN-γ production and tumour burden were found. A smaller proportion of melanoma patients (11 out of 42) compared to healthy volunteers (11 out of 13) exhibited T cell reactivity to CSPG4 [52]. LPS has been reported to induce NG2/CSPG4 in rat microglia cells. Silencing NG2 RNA in LPS-treated microglia blocked the expression of nitric oxide synthase and pro-inflammatory cytokines, including IL-1β and tumour necrosis factor α (TNF-α), but not chemokines like monocyte chemoattractant protein 1 (MCP-1) and stromal cell-derived factor 1 α (SDF-1α) [56]. This suggests that NG2, induced upon microglial stimulation, likely regulates the expression of pro-inflammatory cytokines.

Studies have also reported interactions between chondroitin sulfate (CS) chains of proteoglycans, their degradation products, and the immune system. Primary human NK cells and macrophages secrete CSPGs as metabolic products, and CSPG4 secretion increases following lipopolysaccharide (LPS) stimulation. Splenocytes from ovalbumin (OVA)-immunised mice cultured with CS secreted higher levels of pro-inflammatory cytokines such as IFN-γ, IL-2, and IL-12 and lower levels of anti-inflammatory IL-5 and IL-10 [57]. Furthermore, treatment of BALB/c mice with CS and other glycosaminoglycans (GAGs) induced autoimmune conditions like rheumatoid arthritis by recruiting CD4+ T cells [58]. The treatment of murine NK cells with chondroitinase or a proteoglycan biosynthesis inhibitor significantly reduced IFN-γ secretion through interactions with IL-12. CS has also been shown to stimulate monocytes to secrete IL-1β and induce B cell proliferation in vitro via PKC translocation and activation of protein kinase B (PKB/Akt) [59,60]. Human monocyte-derived dendritic cells cultured with CS, hyaluronic acid, ECM components, and granulocyte-macrophage colony-stimulating factor (GM-CSF) differentiate more rapidly than those cultured with GM-CSF and IL-4, suggesting a role for CS in DC maturation [61]. Conversely, CSPG low-molecular weight disaccharide fragments can modulate inflammatory responses, reducing T cell migration and activation in models of autoimmune encephalitis, inflammation-mediated eye neuropathology, and delayed-type hypersensitivity in Balb/c mice [62]. Furthermore, CS isolated from a crustacean with a unique salvation pattern is able to reduce inflammation and modulate murine melanoma cells [63,64].

In summary, chondroitin sulfate proteoglycans (CSPGs), CS carbohydrate chains, and small molecular weight CS degradation products influence the activation, maturation, proliferation, and migration of various immune cell subsets. The specific roles of CSPG4 in cancer immune responses remain largely unexplored, necessitating further research to determine whether CSPG4 interactions can enhance CSPG4-targeted immunotherapy or mitigate negative immunomodulatory effects.

## 5. CSPG4 as a Target for Cancer Immunotherapy

**Figure 3:**
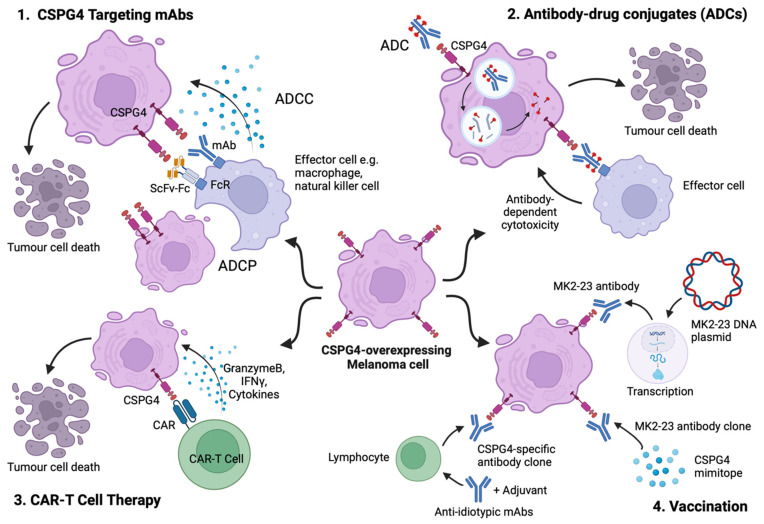
Key cancer antibody immunotherapy strategies targeting CSPG4. 1. CSPG4-targeting antibody therapies induce antibody-dependent cellular cytotoxicity/phagocytosis (ADCC/ADCP), mediated by immune effector cells such as macrophages and natural killer (NK) cells. 2. CSPG4-targeting ADCs deliver cytotoxic drugs directly to the target antigen-expressing cells to induce cell death, while it is possible that the antibody itself can trigger antibody-dependent cytotoxicity mediated by immune effector cells. 3. CAR T cell therapy targeting CSPG4 in melanoma involves engineering T cells to express chimeric antigen receptors (CARs) that specifically recognize and bind to the CSPG4 antigen on the melanoma cells, enabling the T cells to directly the cancer cells. 4. Key vaccination strategies to raise immunity against CSPG4-expressing cancer: (a) Anti-idiotypic monoclonal antibodies that recognise the CSPG4-binding sites (idiotypic regions) on a CSPG4-specific antibody, hence structurally mimicking epitopes of CSPG4, are delivered with adjuvants (aluminum or TLR agonists) to induce an adaptive immune response, promoting CSPG4-specific antibody secretion; (b) DNA vaccines encoding MK2-23 single-chain antibodies trigger both humoral and cellular immune responses against cancer cells; (c) CSPG4 mimotopes, peptides that mimic an epitope on the CSPG4 structure, often coupled with an immunogen such as tetanus toxoid or albumin binding protein are designed to stimulate a humoral immune response against CSPG4-expressing cancer cells. The figure was created with BioRender.com.

The selection of a suitable cancer-associated antigen for monoclonal antibody treatment requires both high expression in the intended target cells and limited expression in non-malignant tissues to ensure sufficient tumour specificity and enhance the therapeutic window. Characterisation of CSPG4 molecular structure, along with its complex role in melanoma oncogenesis, have prompted translational and clinical endeavours to develop targeted immunotherapies. Here, we summarise different approaches, including their clinical applications and ongoing research directions, focusing on CSPG4 immunotherapy for melanoma (Figure 3).

### 5.1. Targeting CSPG4 Using Monoclonal Antibodies

The first monoclonal antibodies against human melanoma-associated antigens were generated and characterised over 50 years ago [65]. Remarkably, the production of these antibodies still relies on isolation from mouse ascitic fluid followed by ammonium sulphate and caprylic acid precipitation [45,53]. Manipulation of mouse-derived antibodies to generate equivalents of the same clone with human Fc regions is necessary for mechanistic studies in human cancer and for ultimate clinical translation.

The anti-tumour effect of mAbs targeting CSPG4 was first reported in severe combined immunodeficiency (SCID) mice injected with human melanoma cell lines [66]. This demonstrated that administration of a murine anti-CSPG4 IgG1 mAb (clone 225.28) significantly reduced tumour size, along with altering the expression of melanogenesis- and metastasis-associated genes. Another CSPG4 targeting murine IgG2a antibody, clone 9.2.27, which recognizes a distinct and spatially distant epitope of human CSPG4, was evaluated as a potential treatment against a human melanoma cell line [67]. This anti-CSPG4 antibody clone demonstrated the ability to function via its Fab region to reduce viability and inhibit colony formation of CSPG4-expressing human melanoma cells, as well as to limit the invasive capacity of a melanoma-derived 3D spheroid. Furthermore, the 9.2.27 IgG clone linked to the chemotactic peptide fMet-Leu-Phe showed early signs of chemotactic activity and macrophage infiltration in preclinical studies. This chemotactic conjugate was later tested in patients with melanoma and colorectal cancer in a phase 1 clinical trial, reporting treatment to be well tolerated [68]. Interestingly, the anti-invasive effect exerted by this CSPG4-specific antibody was not enhanced by the combined treatment of the BRAF-inhibitor PLX4032. These observations were in contrast to results from an earlier study, which demonstrated synergistic effects of PLX4032 with the murine 225.28 IgG1 antibody on inhibiting the growth of melanoma cells in vitro [53]. These contrasting results might be due to employing melanoma cell lines with differential CSPG4 expression, variable susceptibility to BRAF inhibition, or the use of different antibody clones and isotypes. Further functional characterisation of different antibody clones and studies against a range of melanoma models are required. None of these studies have investigated the Fc-mediated functions of different antibody clones in the presence of human immune effector cells.

In addition to full-length mAbs, another study generated and characterised a CSPG4-specific single-chain variable fragment (scFv) fused with a human IgG1 Fc region (scFv-Fc). This compound was studied in both a human melanoma cell line and a human melanoma xenograft model [69]. The CSPG4-specific scFv-Fc antibody reduced phosphorylation of both ERK1/2 and FAK by over 60% in CSPG4-positive cell lines, including melanoma, in the absence of immune effector cells, suggesting the suppression of melanoma growth through direct, Fab-mediated effects. Furthermore, in a human melanoma xenograft model grown in severely immunocompromised (SCID) mice, the scFv-Fc antibody significantly extended mouse survival and inhibited lung metastasis with no detectable adverse effects. 

The importance of evaluating the antibody Fc region with regard to anti-tumour efficacy was highlighted in several studies which evaluated anti-CSPG4 antibodies with human Fc domains, which allowed the study of antibody effector functions in relation to efficacy. One study reported engineering anti-CSPG4 mAbs of the 225.28 murine clone into chimeric antibodies with either IgG1 or IgG4 human constant regions [70]. The anti-CSPG4 IgG1 constructs demonstrated the ability to mediate substantially effective antibody-dependent cellular phagocytosis (ADCP) in patient-derived monocytes and reduced tumour size in human melanoma xenograft models. In contrast, the IgG4 equivalent failed to induce anti-tumour effector functions and significantly impaired the anti-tumour potency of IgG1, a mechanism that may be associated with inhibiting IgG1-mediated FcγRI activation on human immune effector cells. These findings highlight the importance of considering the impact of the Fc domains and the antibody isotype in therapeutic mAbs design. The crucial role of antibody isotype was also emphasised in a study which evaluated the potential advantages of the IgE class Fc regions in an anti-CSPG4 therapeutic antibody [42]. A recombinant anti-CSPG4 antibody generated with a human IgE Fc backbone triggered greater human macrophage infiltration, induction of pro-inflammatory pathways including TNF, IL-1, IL-12, and IFN, and engendered superior anti-tumour functions in human melanoma xenograft models engrafted with human effector immune cells when compared with the corresponding IgG1. Furthermore, anti-CSPG4 IgE significantly prolonged the survival of patient-derived melanoma xenografts engrafted with autologous patient immune cells, showcasing the potency of CSPG4-specific IgE mAbs against CSPG4-expressing patient-derived melanomas. In addition, in vivo safety assessments of a surrogate anti-CSPG4 rat IgE orthologue in an immunocompetent rat model revealed only transient, mild to moderate adverse effects upon repeated antibody administration at high doses, complementing the preclinical evaluation of CSPG4-targeting mAbs [71]. 

Together, these early preclinical studies have showcased the efficacy and therapeutic potential of various CSPG4-targeting mAbs formats and isotypes, shedding light on this emerging therapeutic approach. Given the encouraging results in utilising CSPG4-specific mAbs, further preclinical evaluation, especially of the Fc region humanised constructs, is warranted.

### 5.2. Antibody–Drug Conjugates (ADCs) Directed at CSPG4-Expressing Melanoma

ADCs, comprising monoclonal antibodies specific for a cancer-associated antigen conjugated to a toxic payload, are a revolutionary novel class of anti-cancer therapeutics. To date, several ADCs, including six against solid tumours, are clinically approved by the FDA [72,73]. Ado-trastuzumab emtansine (Kadcyla^®^) and Trastuzumab deruxtecan (Enhertu^®^), both targeting HER2, are ADCs widely used in the treatment of HER2+ metastatic or locally advanced breast cancer. The latter is also approved for HER2+ gastric cancer and non-small cell lung cancer.

Anti-CSPG4 antibodies have been linked with diverse payloads, including conventional cytotoxic drugs, bacterial toxins, and radioisotopes. Early studies utilised the antibody specificity to CSPG4 to amplify the therapeutic precision of a cytotoxic drug whilst reducing the toxicity of chemotherapy. For example, a CSPG4-specific IgG2 mAb clone conjugated to doxorubicin (DXR) via an acid-sensitive linker enhanced tumour-killing cytotoxicity in vitro and suppressed tumour growth in human melanoma-bearing immunodeficient (nude) mice [74]. In another study, the IgG2a 225.28 mouse clone was conjugated with a different chemotherapeutic agent, methotrexate (MTX) [75]. This ADC demonstrated a 3-fold higher effectiveness at inhibiting melanoma cell growth in vitro than MTX alone and suppressed the growth of human melanoma xenografts in nude BALB/c mice without significant off-target toxicities.

A different approach was to attach a radioisotope, bismuth-213 (^213^Bi), to the mAb clone 9.2.27. The specific cytotoxicity of this ^213^Bi-conjugated mAb was first evaluated in vitro, showing strong antitumoral effects in various CSPG4-expressing human melanoma cell lines [76]. This construct was later tested in a dose-escalating phase I clinical trial in patients with stage IV melanoma as 213Bi-cDTPA-9.2.27 [77,78]. The trial reached an objective partial response rate of 10%, with 40% of patients having stable disease within an 8-week follow-up period and an overall 5-year survival rate of 13%. Unexpectedly, the therapy visibly reduced melanoma lesions in some patients. This was surprising for treatment with alpha-emitting isotopes, which primarily function by suppressing further growth rather than by reducing the size of existing tumours. Additionally, no evidence of toxicity was observed across the administered dose range, offering early indications that this might be a safe and effective treatment for metastatic or late-stage melanoma. The underlying factor for unexpected efficacy requires further investigation to progress into later-phase clinical trials. Unfortunately, later investigations and clinical trials were terminated due to a lack of funding.

An optimised drug–antibody ratio (DAR) is a key factor for the ultimate success of ADCs. To increase the cytotoxic payload attached to each antibody, a group encapsulated cisplatin into a human ferritin nanoparticle cage and conjugated it to the CSPG4-specific murine mAb Ep1 (IgG1) [79]. Nanoparticle encapsulation addresses the issues of chemical instability and poor solubility of cisplatin, as well as minimising cytotoxic side effects and enhancing its clinical relevance. This ADC construct impaired the proliferation of CSPG4-expressing melanoma cells in vitro and limited tumour growth in nude mice harbouring human melanoma xenografts.

A new DNA minor groove-binding agent, pyrridinobenzodiazepine (PDD), was conjugated to a chimeric CSPG4-specific mAb constructed by cloning the variable regions of murine 225.28 IgG2a clone into a human IgG1 backbone [80]. In vitro evaluation showed that a small fraction of PDD conjugated to the ADC and a small dose of the ADC (nano- to picomolar dose range) could restrict CSPG4-expressing melanoma cell growth, impair colony formation and induce apoptosis, highlighting the potent cytotoxicity effect exerted from this construct. Furthermore, the ADC restricted human melanoma xenograft growth at 2mg/kg and induced regression at higher doses (5 mg/kg), with negligible adverse effects and no evidence of therapeutic resistance in the residual tumour. The apparent absence of treatment-induced toxicity or development of treatment resistance reaffirms the potential of this approach to provide future clinical benefit for patients.

Bacterial toxins have also been considered as potential payloads for ADCs. For example, saporin, a type-1 ribosome-inactivating protein, was conjugated to the CSPG4-specific mAb 225.28 mouse clone and tested against melanoma and triple-negative breast cancer cell lines [81]. While saporin is not internalised and thus cannot impact cell viability, conjugation to the CSPG4-specific mAb 225.28 significantly enhanced saporin internalization and cytotoxic effects in vitro. However, the large molecular size of saporin-conjugated mAb makes it a poor candidate for targeting cancer cells in solid tumours due to insufficient penetration of the ADC complex. To overcome this, another study linked another type I ribosome-inactivating protein, gelonin, to the scFv fragment of mAb 225.28 to reduce the size of the treating complex. This smaller conjugate significantly restricted tumour growth in amelanotic melanoma xenografts, supporting the concept of a CSPG4-targeted immunotoxin and the clinical potential of such a construct. In another ex vivo study, a Pseudomonas exotoxin A-conjugated CSPG4-specific scFv triggered antigen-specific apoptosis in over 70% of patient-derived primary melanoma cells [82]. However, the main drawback of this construct is that the bacteria-derived toxin can trigger neutralising antibodies and thus, more rapid drug clearance of the conjugate from the bloodstream. 

Another group generated an scFv fused with soluble human TNF-related apoptosis-inducing ligand (TRAIL), with the aim of triggering pro-apoptotic TRAIL signalling and inhibiting the pro-tumorigenic downstream signalling pathways associated with CSPG4 [83]. Daily treatment with a low dose of this ADC construct resulted in significant growth suppression of human melanoma xenografts in nude mice. The elimination of cancer cells occurred due to a combination of activation of TRAIL-associated apoptotic signalling and dephosphorylation of key proteins such as FAK or Src kinases. Furthermore, synergistic anti-tumour activity was achieved with the co-treatment of the sigma receptor (σR) rimcazole, a σ-ligand known to have selective cytotoxicity in various tumours. The application of a smaller construct, like scFv, has some advantages over full-length antibody–drug conjugates due to better infiltration into solid tumours. However, the lack of an Fc region, and hence of the FcRn and FcR domain of a full-length antibody, would result in a shorter half-life and faster clearance from the circulation, potentially limiting therapeutic efficacy.

Although ADCs are efficient at selectively eliminating melanoma cells and offer low levels of off-target toxicity compared to non-targeted cytotoxic therapeutics, none of these agents has yet reached clinical approval. With the identification of CSPG4 as a target in melanoma and other tumours, optimisation of the antibody, linker, and payload will likely be required as a combined effort to overcome drug resistance, minimise effects, and improve long-term clinical outcomes. Moreover, most preclinical studies of CSPG4-targeting ADCs have been performed on immunodeficient mice, overlooking the Fc-mediated effect of antibodies in such models. Evaluating ADCs in immuno-humanised mice could be valuable in unveiling potential immune-stimulating anti-tumoral mechanisms of both payloads and mAbs.

### 5.3. CSPG4-Specific Chimeric Antigen Receptors (CARs)

Chimeric antigen receptors (CARs) are engineered proteins that, within one receptor, combine the antigen recognition domain of a monoclonal antibody (mAb) with a T cell receptor (TCR) structure and its intracellular co-stimulating components. When these CAR complexes are expressed on the surface of T cells, they can direct the cytotoxic functions of these immune cells to antigen-expressing cancer cells. One of the first published studies of T cells expressing a CSPG4-specific CAR utilised an scFv region based on the mouse IgG1 monoclonal antibody (mAb) clone 225.28. When co-cultured with a melanoma cell line, CSPG4-targeting CAR T cells proliferated, produced cytokines (such as IFN-γ and TNF-α), and specifically lysed CSPG4-expressing cancer cells. The CAR T cells were also shown to react to human melanoma explants. However, in vivo studies were not performed [84]. 

In two different studies, the 763.74 IgG2a mAb mouse clone was engineered into a CAR construct and tested against human melanoma cell lines and human melanoma xenografts in NOD scid gamma (NSG) immunodeficient mice [85,86]. In one of these studies, CARs obtained from this mAb clone did not have anti-tumour activity [85]. However, in the more recent study, CAR T cells produced pro-inflammatory cytokines and exhibited cytotoxic activity when co-cultured with CSPG4-expressing melanoma cells in vitro. Additionally, mice treated with the CAR T cells bore smaller tumours and showed improved survival [86]. The latter report utilised a CAR with a CD20-targeting chimeric co-stimulatory receptor (CCR), which possesses no lone cytolytic potential due to the absence of a CD3ζ activation element. This achieved selective induction of the anti-tumour response upon simultaneous engagement of the CAR and CCR target antigens and thus, the overall functional capacity of the construct [86]. Interestingly, CAR constructs based on three different mAb clones were tested, and the highest cytokine release was achieved using an anti-CSPG4 scFv based on the mouse mAb clone TP41.2 [41]. These findings highlight the importance of each CAR component, including the variable region, the TCR and the co-stimulatory domain, in achieving anti-tumour efficacy. 

Even though CAR T cells have shown promise in preclinical studies, some major obstacles obstruct progression into clinical trials. With CAR T cell therapy, there is a high proportion of severe side effects resulting from on-target/off-tumour toxicities, cytokine release syndrome (CRS), or graft versus host disease when T cells from allogeneic donors are used. Some of those limitations can be overcome by CAR expression limited to different populations of immune cells or specific sub-populations. One such strategy used natural killer T cells (NKT cells) expressing CAR based on the mouse 9.2.27 IgG2 mAb. This construct produced a significant amount of TNF and IFN-γ after co-culture with melanoma cells, but the secretion of these pro-inflammatory mediators was much lower than exhibited by the corresponding CD8+ CAR T cells. Despite this, in vitro cytotoxicity against melanoma cells was comparable between the two CAR subsets [87]. This shows that CAR NKT cells might be less likely to cause CRS while still being able to efficiently lyse cancer cells in an antigen-specific manner. 

Alternatively, CSPG4-CAR T cells produced through mRNA electroporation rather than viral transduction, a technique which reduces toxicity through temporary restriction of CAR expression, resulted in cytokine secretion and successful lysing of CSPG4-positive melanoma cells [88]. However, anaphylaxis was reported in one patient in a clinical trial for an mRNA-CAR T cell therapy targeting the TSA mesothelin, likely due to immune activation from repeated administration of CAR T cells [89]. Furthermore, overcoming the therapeutic limitation posed by TSA heterogeneity and tumour escape via loss of cell surface antigen has been considered by targeting multiple MAAs (e.g., CSPG4 alongside tyrosinase-related protein 1). For these approaches, synthetic agnostic receptor (SAR)-expressing T cells are activated once bispecific antibodies bind to both the SARs and TSAs [90,91]. This construct demonstrated antitumour activity and prolonged long-term survival in various cell line- and patient-derived xenograft models of melanoma [91]. Moreover, the use of T cell receptors (TCRs) instead of CARs to target CSPG4 has addressed difficulties in T cell expansion in vitro [92]. Anti-CSPG4 TCRs have shown T cell recognition of CSPG4-expressing melanoma cell lines [92,93]. However, anti-CSPG4 TCRs may be more susceptible to on-target/off-tumour toxicities than their CAR T cell counterparts.

Similar results were obtained when the same mAb clone was used to construct a CAR in γ/δ T cells, a T cell subset that can directly recognise cancer cells without antigen presentation on major histocompatibility complex (MHC). The study showed overall lower levels of cytokine release by γ/δ CAR T cells compared to the corresponding CD8+ CAR T cells but a similar amount of cancer cell lysis. Importantly, in contrast to CD8+ CAR T cells, γ/δ T cells did not release cytokines in co-cultures with cells not expressing CSPG4. This suggests that CAR γ/δ T cells are potentially less active in a non-antigen-specific manner by driving specific cytotoxic effects only towards CSPG4-expressing cells [94]. Moreover, both NKT and γ/δ T cells could have the benefit of being obtained from healthy donors as they do not cause alloreactivity, reducing the risk of graft versus host disease. Results of these studies, reporting the potential for CAR NKT and γ/δ T cells, require validation in animal models to assess the effectiveness of such CAR constructs in vivo as well as to evaluate the occurrence of toxicities such as CRS. 

Another strategy for improving the efficiency of CARs is combining them with another receptor on the surface of a T cell. To decrease the possibility of on-target/off-tumour toxicity, one group combined a low-affinity CSPG4-CAR (225.28 clone-derived scFv linked to an IgG4 Fc region) with a chimeric co-stimulatory co-receptor (CCR) directed at CD20. The T cells expressing both receptors were very efficient at limiting the growth of human melanoma xenografts grown in mice, while the low-affinity CAR alone showed only weak anti-tumour effects. The authors suggested that the T cells expressing both receptors will selectively lyse tumour cells because of simultaneous activation with CSPG4 expressed on the tumour and CD20 expressed either on the tumour or on bystander/tumour-infiltrating B cells [95]. Such simultaneous activation is much less likely to occur in healthy tissues. This hypothesis, however, was not tested by evaluating the cytotoxicity of those CAR T cells towards healthy, non-melanoma CSPG4-expressing cells. Nonetheless, this construct could have the advantage of specifically targeting melanoma cells with stem cell-like features, which are known to express CD20 and play a role in tumour initiation and progression [96]. 

Alternatively, combining CSPG4-CAR T cells with conventional treatments for melanoma, like BRAF and MEK inhibitors (BRAFi/MEKi), may provide a further treatment strategy. One study evaluated the effects of co-culturing CAR-transfected CD8+ T cells with clinically relevant concentrations of variations of BRAFi/MEKi. Dabrafenib (BRAFi) and Trametinib (MEKi) did not impair the cytolytic capacity of CSPG4-CAR T cells and induced moderate cytokine secretion [97]. Collectively, targeting melanoma with CPSG4-CAR T cells with BRAFi/MEKi may be optimised to have synergistic effects on tumour regression. 

Despite efficacy in patients with haematological cancers, CAR T cell therapies in solid tumours, including melanoma, require further optimisation. Major obstacles include poor T cell infiltration into tumour tissues and an immunosuppressive tumour microenvironment in solid cancers that reduces CAR T cell efficacy. This may be why, despite promising data from studies in vitro, data in vivo and in clinical trials have been limited. To date, only one CSPG4-targeted CAR T cell construct has progressed to phase 1/2 clinical trial (ClinicalTrials.gov, NCT06096038) for patients with head and neck squamous cell carcinoma (HNSCC). Since this trial is at the recruiting stage, no clinical data is currently available. In future, if promising efficacy and safety data can be derived from this trial, this can potentiate the translation of this, and other CAR T cell immunotherapies focused on CSPG4 for the treatment of melanoma and other tumors.

### 5.4. Anti-Idiotypic and Mimotope Vaccines Inducing Humoral Responses against CSPG4

Anti-idiotypic antibodies target the binding sites (idiotypic regions) on an antigen-specific antibody through structural mimicry of the target antigen. When introduced into a patient as a vaccine, they stimulate the immune system to produce antibodies that can recognise and react with the target antigen. This strategy has been applied to CSPG4, as described below. 

An early phase I clinical trial where an anti-idiotypic mAb MF11-30 against the 225.28 CSPG4-specific mAb clone was administered to 21 patients with stage IV melanoma reported complete remission in one patient and minor response in three other patients. No toxicities or allergic reactions were reported across a range of doses [98]. Later attempts at utilising anti-idiotypic mAbs in a phase I trial of 25 patients with stage IV melanoma employed the MK2-23 anti-idiotypic mAb, which recognises the 763.74 anti-CSPG4 clone. The anti-idiotypic antibody was administered together with Bacille Calmette–Guérin (BCG) to serve as an adjuvant and thus trigger an adaptive immune response. In this study, 56% of patients developed anti-CSPG4 antibodies, and three of them achieved a partial response. Importantly, the overall survival of the patients who developed antibodies as a result of the therapy was significantly longer than those who did not develop humoral anti-CSPG4 immunity. The treatment caused significant side effects at the site of injection, which were most likely associated with BCG administration [99]. Safety issues associated with the use of BCG as an adjuvant in the clinic prevented MK2-23 from progressing further [100]. To overcome these issues, the MK2-23 variable regions were covalently linked to a constant human immunoglobulin region, and the construct was conjugated with human interleukin 2 (IL-2) to help potentiate an immune response. Immunisation with this fusion antibody construct elicited stronger humoral and cellular responses in BALB/c mice than immunisation with the same MK2-23 mAb conjugated to keyhole limpet hemocyanin (KLH) as a carrier and IL-2 administered separately. KLH is an immunogenic carrier protein that increases antigenic immune responses to haptens and other weak antigens, and this therefore, suggests that IL-2 could serve as an alternative to KLH, which is difficult to standardise for clinical use [100].

Another group tested DNA vaccines encoding MK2-23 scFv as an alternative to administering mAb MK2-23. C57BL/6 mice were challenged subcutaneously with B16 melanoma cells that do not express CSPG4. The DNA-immunised mice had a delayed onset of melanoma and bore smaller tumours than the ones injected with a control plasmid. Since these melanoma cells do not express CSPG4, a suspected mechanism of action of this DNA vaccination was through triggering an immune response targeting the CSPG4-expressing pericytes in tumour blood vessels [101]. This study demonstrated that DNA vaccines could potentially be an effective alternative to anti-idiotypic antibody vaccination, although the mechanism by which such a therapy could target CSPG4-negative tumours requires further investigation.

Another strategy for the development of anti-cancer vaccines is using mimotopes, peptides that mimic an epitope on CSPG4, to induce a polyclonal antibody response against this antigen. One study utilised a CSPG4 mimotope recognised by the 225.28 mAb clone coupled to tetanus toxoid as an immunogen, which was administered in rabbits. The vaccination-induced antibodies that cross-reacted with native rabbit CSPG4. Antibodies from the immunised rabbits inhibited the growth of melanoma cells in vitro and mediated the lysis of melanoma cells in an ADCC assay in the presence of effector human PBMCs. Direct, Fab-mediated growth inhibition also occurred in most CSPG4-expressing cancer cells in the absence of effector cells [102]. Similar results were obtained when mimotopes of the epitope recognised by the 225.28 mAb clone were fused to a different immunogenic carrier, albumin binding protein. Vaccination in BALB/c mice induced the production of antibodies that recognise melanoma cells in vitro. Moreover, the antibodies caused tumour-specific lysis of about 9% of human melanoma target cells in an ADCC assay in the presence of murine immune effector cells [103]. However, neither vaccination approach, using mimotopes recognised by the 225.28 antibodies, was tested in vivo. 

A different group tested immunisation with a peptide mimicking an epitope on CSPG4 recognised by the 763.74 mAb clone in rabbits. They observed that CSPG4-specific antibodies isolated from vaccinated rabbits triggered ADCC of cancer cells in the presence of human PBMCs but not complement-dependent cytotoxicity (CDC) in the presence of human serum. The effectiveness of vaccination with mimotopes recognised by the 763.74 mAb remains to be tested in vivo [104].

The anti-idiotypic antibody or mimotope vaccine strategies have not been the focus of recent research. Nevertheless, these early studies show that vaccines inducing a humoral response against CSPG4 could hold promise for the treatment of melanoma, with longstanding effects, minimal toxicities and limited development of resistance [33]. Moreover, one approach to improve the efficacy of cancer vaccines is to introduce adjuvants, such as aluminium and Toll-like receptor (TLR) agonists. Aluminium was shown to improve antibody stability, elicit immune responses and show synergistic anti-tumour effects with the anti-idiotypic antibody vaccine racotumomab in patients with non-small cell lung cancer [105,106,107]. Furthermore, the TLR agonist imiquimod, which activates innate immunity, promoted immune cell infiltration and T cell responses in combination with a multi-peptide cancer vaccine in a patient with metastatic melanoma [108]. 

Recent developments in vaccine design and translation to the clinic have been precipitated by the application of mRNA vaccine technologies during the COVID-19 pandemic. FixVac (BNT111) is a liposomal RNA (RNA-LPX) vaccine that targets NY-ESO-1 (New York oesophageal squamous cell carcinoma 1), MAGE-A3 (melanoma-associated antigen A3), tyrosinase, and TPTE (transmembrane phosphatase with tensin homology). These vaccine targets are non-mutated melanoma-associated antigens that show high and prevalent expression in melanoma, restricted expression and distribution in non-malignant tissues, and immunogenicity, characteristics that are shared with CSPG4. This vaccine has been translated to a first-in-human clinical trial in advanced melanoma in combination with PD1 inhibition, with encouraging clinical responses in heavily pre-treated patients and signs of activation of adaptive immune responses (Lipo-MERIT trial, ClinicalTrials.gov identifier NCT02410733) [109]. 

Current advances in cancer vaccines and vaccine adjuvants have not been applied to CSPG4. However, it is conceivable that based on the high expression and prevalence of CSPG4 in melanoma at all disease stages and its restricted distribution in normal tissues, future research will likely incorporate CSPG4 as a target for novel vaccine approaches.

## 6. Conclusions and Future Perspectives

Despite the emergence of novel immunotherapies, such as ICIs, in treating melanoma, therapies that engage immune responses directly against melanoma cells are lacking. Identification and validation of a CSPG4-targeting approach could potentially address the unmet needs of patients who do not respond to ICIs and BRAF/MEK inhibitors, as well as those who develop secondary resistance. CSPG4 has many attributes of an appealing drug target. It is overexpressed on the surface of cancer cells but exhibits low or restricted expression in non-malignant tissues. Additionally, strong evidence suggests it is also a functional oncogene with diverse roles in in cancer progression and dissemination. Given the immunogenic nature of melanoma, it is possible that immunotherapies may tap into anticipatory heightened immune surveillance, marking melanoma cells for immune clearance. In support of this notion, several treatments specifically targeting CSPG4, including mAbs, CAR T, NKT and γ/δ T cell constructs, ADCs, and anti-idiotypic or mimotope vaccination strategies have shown some success in targeting melanoma in vitro and in vivo in a wide range of preclinical models and early clinical trials. Yet, few of these therapies have progressed into testing in later-phase human trials, and none are presently being evaluated in patients. 

A multifaceted approach is needed to realise the potential of CSPG4-based therapeutics in the clinic. First, a greater understanding of the role of CSPG4 in melanoma is required. Significant gaps remain in our understanding of CSPG4 regulation and its contributions to melanoma growth, dissemination, and evasion. There is a paucity of information on the immunogenicity of CSPG4, particularly given immune suppression in the tumour microenvironment is a known hindrance to immunotherapy [11,29]. Advancing knowledge in this area will provide pivotal clues for designing the next generation of CSPG4-based precision therapeutics that successfully engage anti-tumour immunity. Secondly, understanding the tumour immune microenvironment and the expression, localization and distribution not only of CSPG4-expressing cancer cells but also co-expression and co-localization with immune effector cells, Fc receptors, and checkpoint molecules are all critical for guiding antibody and immunotherapy design. Thirdly, comprehensive preclinical studies which more aptly reflect human disease and human immunity will help guide productive antibody engineering approaches and the selection of mAbs of different formats and isotypes. Given the vast differences in FcR expression and distribution between rodents and humans, the design and testing of antibodies with human Fc regions will be pivotal in generating compounds that are relevant, functional and applicable in human clinical settings. Adapting humanised mouse models would also allow evaluation of CSPG4-targeted immunotherapy, with a focus on specific immune cell subtypes such as tumour-associated macrophages [110].

Additionally, studying CSPG4 expression at different disease stages, including residual or recurrent disease following treatment, will help select patient groups that benefit the most from CSPG4 therapeutics. Furthermore, transitioning into the next stage of research with clinical evaluation of the safety and efficacy of anti-CSPG4 agents is timely, as few compounds have reached the clinical trial stage. Those which have progressed, such as MK2-23, have involved small sample sizes and failed to demonstrate persistent improved survival for many patients [99]. In addition to demonstrating efficacy, preventing and minimising any toxicities of CSPG4-directed treatments is paramount. Future strategies will likely include treatment combinations to further bolster efficacy. One such example may be transmembrane-targeting peptides which disturb CSPG4 function or enhance the effectiveness of CSPG4-specific CAR T cells against melanoma. This may be achieved by engineering T cells to co-express chemokine or cytokine receptors [29,111,112]. This has been shown for GD2-targeted CAR T cells that demonstrate promising anti-tumour cytotoxic profiles against melanoma, and in which the co-expression of CCR2b or the IL-7 receptor (NCT03635632) can augment infiltration and persistence of these cells at the solid tumour sites [111,113,114]. In principle, this can be applied to CSPG4-specific CAR T cells to enhance their antitumour functions.

Emerging, exciting avenues of research in immunotherapies include strategies such as nanoparticles that can sequester toxic components from the bloodstream to target melanoma cells in tissues [79]. IgE antibodies may also emerge as a future immunotherapy avenue for melanoma, based on the high affinity of IgE towards its Fcε receptors on immune effector cells, the absence of known inhibitory receptors and the ability of IgEs to recruit and stimulate tumour-associated macrophages and pro-inflammatory pathways in a manner beyond conventional IgG therapeutic antibodies. In addition, tri-specific killer engagers (TriKEs) are emerging as promising immunotherapy to boost the ADCC function of natural killer (NK) cells against tumor cells [115]. In future, TriKE designed with CD16-specific (to engage NK cells) and CSPG4-specific (to target melanoma cells) scFvs, linked by a peptide incorporating the cytokine IL-15 to stimulate NK cells, could potentially complement traditional antibody therapies and further enhance antitumor responses.

Beyond melanoma, CSPG4-based immunotherapeutics could have wider application in the treatment of other cancers such as triple-negative breast cancer (TNBC), glioblastoma, and particularly cutaneous squamous cell carcinomas (SCC) [41,86,116]. Recent evidence from gene expression studies indicates a potential involvement of CSPG4 in squamous cell carcinoma (SCC) [117]. A recent study showed that CSPG4 is expressed on the tumour/stroma interface in the recessive dystrophic epidermolysis bullosa (RDEB)-SCC, contributing to EMT transition and tumour invasion via TGFβ signaling [118]. The current absence of a widely accepted diagnostic marker presents a challenge for clinicians caring for patients with SCC, highlighting this as an area of substantial opportunity to improve outcomes. This issue is especially critical for elderly or frail patients and individuals with conditions like epidermolysis bullosa (EB), where a delayed diagnosis often has life-threatening consequences. A better understanding of the structure and tissue-specific expression of CSPG4 will help determine its utility as both a diagnostic marker and therapeutic target across a wide range of solid malignancies, where it is uniquely poised to benefit patients with significant unmet needs.
